# Immunomodulatory, antioxidant, and growth-promoting properties of *Avicennia marina* leaf extract on Nile tilapia

**DOI:** 10.1038/s41598-025-30685-z

**Published:** 2025-12-17

**Authors:** Mohammed F. El Basuini

**Affiliations:** 1 Animal Production Department, Faculty of Agriculture, Tanta University, Tanta, 31527 Egypt; 2 Faculty of Desert Agriculture, King Salman International University, El Tor, South Sinai 46612 Egypt

**Keywords:** Cytokine expression, Digestive enzymes, Innate immunity, Mangrove, Stress biomarkers, Biochemistry, Immunology, Physiology, Zoology

## Abstract

This study evaluated the effects of *Avicennia marina* leaf aqueous extract on Nile tilapia performance. Fish (5.15 g, *n* = 675) were fed diets containing 0, 100, 200, 300, and 400 mg/kg extract for 60 days. Fish fed 200–300 mg/kg diets showed better growth status (*P* < 0.05), along with improved feed utilization. Digestive enzyme levels (amylase, lipase, protease) were better, and intestinal histomorphology was optimized, particularly at 200–300 mg/kg. Biochemical assessment of the serum indicated increased levels of total protein, albumin, and globulin, with reduced glucose, cholesterol, and cortisol in fish receiving 200–400 mg/kg. Hepatic antioxidants (SOD, CAT, GSH-Px) increased, while malondialdehyde (MDA) decreased. Immune responses (lysozyme, NBT reduction, *Streptococcus agalactiae* inhibition) were enhanced, and gene expression showed upregulated *IGF-1*, *SOD*, *CAT*, and *GSH-Px* with downregulated *IL-1β* and *TNF-1α* in the 200–400 mg/kg groups. Dietary *A. marina* extract at 200–300 mg/kg enhanced growth, digestive efficiency, antioxidant status, immune functions, and gene expression in *Oreochromis niloticus*, suggesting its promise as a natural feed additive in aquaculture.

**Supplementary Information:**

The online version contains supplementary material available at 10.1038/s41598-025-30685-z.

## Introduction

Aquaculture has seen significant expansion in recent decades, fueled by the increasing global demand for fish protein, which plays a crucial role in nutrition and food security^1^. Nile tilapia (*Oreochromis niloticus*), one of the most widely cultured freshwater fish species worldwide, plays a pivotal role in meeting this demand because of its rapid growth, adaptability, and high nutritional value^2^. However, the intensification of fish farming has introduced several challenges, including physiological stress, impaired immune function, and increased vulnerability to infectious diseases, which can collectively hinder sustainable production^3^.

Historically, antibiotics have been employed extensively to prevent disease outbreaks and enhance productivity. Nevertheless, the widespread use of antibiotics raises significant concerns, such as the emergence of antibiotic-resistant pathogens, residual contamination in aquatic products, and detrimental effects on aquatic ecosystems^4^. These issues emphasize the urgent need for sustainable and eco-friendly alternatives that support fish health and improve aquaculture sustainability.

In this regard, natural plant-derived bioactive compounds have gained attention as promising feed additives, providing antioxidant, immunostimulatory, and stress-alleviating properties^5,6^. Unlike synthetic chemicals, these plant-derived supplements are biodegradable, environmentally benign, and align with the principles of sustainable aquaculture practices^7,8^. Among various plants, mangrove species, particularly *Avicennia marina*, have garnered attention due to their rich phytochemical content, including flavonoids, polyphenols, tannins, and saponins, which contribute to their notable antimicrobial, antioxidant, and immunomodulatory activities^9–11^.

Despite the extensive medicinal and pharmacological applications of *A. marina* extracts in terrestrial systems^12–14^, their potential in aquaculture has been only limitedly investigated^15^. Leveraging the bioactive properties of *A. marina* aqueous leaf extract could offer a natural strategy to augment growth, digestive efficiency, antioxidant defenses, and immune responses in aquatic organisms, reducing reliance on antibiotics and mitigating stress-induced health challenges.

From a practical standpoint, the implementation of *Avicennia marina* extract in commercial aquaculture appears economically feasible due to the species’ broad natural distribution and the abundance of mangrove resources. *A. marina* is one of the most widely distributed mangrove species globally, thriving along tropical and subtropical coastlines that extend from East Africa and the Arabian Peninsula to the Indo-Pacific region^16^. Globally, mangrove forests occupy approximately 15.2 million ha (about 152,000 km²) across 118 countries, providing substantial renewable biomass resources^17^. Mangrove leaves, particularly those of *A. marina*, represent a renewable and sustainable bioresource that can be utilized without compromising ecosystem integrity when responsible management and harvesting practices are applied^16,18^. These forests are already recognized for their socio-economic value, supporting fisheries, livestock feed, and other livelihood applications under sustainable management schemes^16^. The relatively low dietary inclusion levels required for effective supplementation (250–300 mg/kg diet) further enhance the practicality and economic viability of *A. marina* use in aquaculture^15^. When combined with its renewable availability and proven bioactive potential, *A. marina* offers a cost-effective, sustainable alternative to synthetic immunostimulants and growth promoters currently employed in aquafeed formulations.

Therefore, this study aims to evaluate the effects of dietary supplementation with *Avicennia marina* leaf aqueous extract on the growth performance, digestive efficiency, antioxidant capacity, and immune responses of Nile tilapia (*Oreochromis niloticus*).

## Materials and methods

### Collection and Preparation of mangrove leaf extract

Mangrove leaves (*Avicennia marina*) were sourced from Ras Mohamed, Egypt (27.727792°N, 34.247520°E). After collection, the leaves were air-dried under shade conditions and subsequently finely pulverized. The aqueous extract was produced using an infusion technique. The aqueous extract was prepared using an infusion technique^19^. Specifically, the powdered leaves were immersed in distilled water at a ratio of 1:2 (w/v) for 24 h. After the infusion process, the mixture was filtered (Whatman No. 1 filter paper) using a suction system connected to a rotary evaporator maintained at 40 °C. The concentrated extract obtained was placed in sterile tubes and kept at 4 °C for subsequent chemical analysis. The chemical profile of the *A. marina* leaf aqueous extract was examined using a Trace GC Ultra/ISQ Single Quadrupole Mass Spectrometer (Thermo Scientific, USA), fitted with a TG-5MS fused silica capillary column (30 m × 0.25 mm × 0.1 μm film thickness). The oven temperature began at 150 °C and was held for 4 min, then increased to 280 °C at a rate of 5 °C per minute, where it was maintained for another 4 min. The injector and mass spectrometry transfer line were both set to 280 °C, while helium was used as the carrier gas at a steady flow rate of 1 mL/min. Mass spectra were generated via electron ionization (70 eV), and the components present in extract were determined by comparing their retention times and spectral data against established databases (NIST and WILEY).

## Experimental design

Nile tilapia were obtained from a private aquaculture facility in Kafr Elsheikh Governorate and transported to the Desert Agriculture Research Unit at KSIU, Egypt, where the feeding trial was conducted. After arrival, the fish were acclimated for 14 days. Subsequently, they were distributed into 15 circular tanks (200 L), with each tank containing 45 fish (initial weight: 5.15 ± 0.21 g). The feeding experiment spanned 60 days, following a 12-hour light/dark cycle with constant aeration. Key water parameters were consistently monitored, including temperature (26.12 ± 0.26 °C), dissolved oxygen (6.75 ± 0.21 mg/L), pH (7.61 ± 0.24), and total ammonia (0.02 ± 0.001 mg/L). Fish were manually fed to apparent satiation three times each day, at 7:00 A.M., 1:00 P.M., and 7:00 P.M. The experimental diets comprised a control diet and four diets supplemented with mangrove leaf aqueous extract at 100, 200, 300, and 400 mg/kg, as recommended by El Basuini et al.^15^. All diets were designed to provide 31.26 ± 0.34% crude protein and 8.28 ± 0.14% crude fat (Table 1), following previously recommendations^20^.

**Table 1:** Composition and nutritional profile of the basal diet (%, dry matter basis; *n* = 3).

Ingredient	Inclusion (%)	Chemical profile	%
Corn grains	38.25	Crude protein	31.26 ± 0.34
Corn gluten meal	6.00	Crude lipid	8.28 ± 0.14
Poultry meal	6.00	Crude Fiber	2.56 ± 0.09
Fish meal	17.00	Ash	6.88 ± 0.11
Soybean meal	21.00		
Rice bran	5.00		
Soybean oil	3.50		
Calcium carbonate	1.00		
Common salt	0.25		
Premix ^a^	2.00		
Total	100		

^a^ The composition of the premix is described in detail by Ibrahim et al.^20^.

### Performance metrics

Following the 60-day feeding period, fish performance was evaluated through various metrics. The initial (W0 day) and final body weight (W60 day), total length (TL), and the weights of the viscera (VW), liver (LW), and intestines (IW) were recorded. These measurements were used to calculate the next indices:

$$\:\mathrm{W}\mathrm{e}\mathrm{i}\mathrm{g}\mathrm{h}\mathrm{t}\:\mathrm{g}\mathrm{a}\mathrm{i}\mathrm{n}\:\left(\mathrm{W}\mathrm{G},\frac{\mathrm{g}}{\mathrm{f}\mathrm{i}\mathrm{s}\mathrm{h}}\right)\hspace{0.17em}=\hspace{0.17em}\mathrm{W}60\:\mathrm{d}\mathrm{a}\mathrm{y}\:\--\:\mathrm{W}0\:\mathrm{d}\mathrm{a}\mathrm{y}$$

$$\:\mathrm{S}\mathrm{p}\mathrm{e}\mathrm{c}\mathrm{i}\mathrm{f}\mathrm{i}\mathrm{c}\:\mathrm{g}\mathrm{r}\mathrm{o}\mathrm{w}\mathrm{t}\mathrm{h}\:\mathrm{r}\mathrm{a}\mathrm{t}\mathrm{e}\:(\mathrm{S}\mathrm{G}\mathrm{R},\:\mathrm{\%}/\mathrm{d}\mathrm{a}\mathrm{y})=\frac{\mathrm{L}\mathrm{n}\:{\mathrm{W}}_{60\:\mathrm{d}\mathrm{a}\mathrm{y}}-\mathrm{L}\mathrm{n}\:{\mathrm{W}}_{\mathrm{O}\:\mathrm{d}\mathrm{a}\mathrm{y}}}{60}\times\:100$$

$$\:\mathrm{S}\mathrm{u}\mathrm{r}\mathrm{v}\mathrm{i}\mathrm{v}\mathrm{a}\mathrm{l}\:\mathrm{r}\mathrm{a}\mathrm{t}\mathrm{e}\:\left(\mathrm{S}\mathrm{R},\:\mathrm{\%}\right)=\frac{\mathrm{F}\mathrm{i}\mathrm{n}\mathrm{a}\mathrm{l}\:\mathrm{f}\mathrm{i}\mathrm{s}\mathrm{h}\:\mathrm{c}\mathrm{o}\mathrm{u}\mathrm{n}\mathrm{t}}{\mathrm{I}\mathrm{n}\mathrm{i}\mathrm{t}\mathrm{i}\mathrm{a}\mathrm{l}\:\mathrm{f}\mathrm{i}\mathrm{s}\mathrm{h}\:\mathrm{c}\mathrm{o}\mathrm{u}\mathrm{n}\mathrm{t}}\times\:100$$

$$\:\mathrm{F}\mathrm{u}\mathrm{l}\mathrm{t}\mathrm{o}\mathrm{n}' \mathrm{s}\:\mathrm{c}\mathrm{o}\mathrm{n}\mathrm{d}\mathrm{i}\mathrm{t}\mathrm{i}\mathrm{o}\mathrm{n}\:\mathrm{f}\mathrm{a}\mathrm{c}\mathrm{t}\mathrm{o}\mathrm{r}\:\left(\mathrm{K}\:\mathrm{f}\mathrm{a}\mathrm{c}\mathrm{t}\mathrm{o}\mathrm{r}\right)=\frac{\mathrm{B}\mathrm{o}\mathrm{d}\mathrm{y}\:\mathrm{w}\mathrm{e}\mathrm{i}\mathrm{g}\mathrm{h}\mathrm{t}}{{\mathrm{L}\mathrm{e}\mathrm{n}\mathrm{g}\mathrm{t}\mathrm{h}}^{3}}\times\:100$$

$$\:\mathrm{F}\mathrm{e}\mathrm{e}\mathrm{d}\:\mathrm{c}\mathrm{o}\mathrm{n}\mathrm{v}\mathrm{e}\mathrm{r}\mathrm{s}\mathrm{i}\mathrm{o}\mathrm{n}\:\mathrm{r}\mathrm{a}\mathrm{t}\mathrm{i}\mathrm{o}\:\left(\mathrm{F}\mathrm{C}\mathrm{R}\right)=\frac{\mathrm{F}\mathrm{e}\mathrm{e}\mathrm{d}\:\mathrm{I}\mathrm{n}\mathrm{t}\mathrm{a}\mathrm{k}\mathrm{e},\:\mathrm{g}}{\mathrm{W}\mathrm{G},\:\mathrm{g}}$$

$$\:\mathrm{H}\mathrm{e}\mathrm{p}\mathrm{a}\mathrm{t}\mathrm{o}\mathrm{s}\mathrm{o}\mathrm{m}\mathrm{a}\mathrm{t}\mathrm{i}\mathrm{c}\:\mathrm{i}\mathrm{n}\mathrm{d}\mathrm{e}\mathrm{x}\:\left(\mathrm{H}\mathrm{S}\mathrm{I},\:\mathrm{\%}\right)=\frac{\mathrm{L}\mathrm{V},\:\mathrm{g}}{{\mathrm{W}}_{60\:\mathrm{d}\mathrm{a}\mathrm{y}},\:\mathrm{g}}\times\:100$$

$$\:\mathrm{V}\mathrm{i}\mathrm{s}\mathrm{c}\mathrm{e}\mathrm{r}\mathrm{o}\mathrm{s}\mathrm{o}\mathrm{m}\mathrm{a}\mathrm{t}\mathrm{i}\mathrm{c}\:\mathrm{i}\mathrm{n}\mathrm{d}\mathrm{e}\mathrm{x}\:\left(\mathrm{V}\mathrm{S}\mathrm{I},\:\mathrm{\%}\right)=\frac{\mathrm{V}\mathrm{W},\:\mathrm{g}}{{\mathrm{W}}_{60\:\mathrm{d}\mathrm{a}\mathrm{y}},\:\mathrm{g}}\times\:100$$

$$\:\mathrm{I}\mathrm{n}\mathrm{t}\mathrm{e}\mathrm{s}\mathrm{t}\mathrm{i}\mathrm{n}\mathrm{o}\mathrm{s}\mathrm{o}\mathrm{m}\mathrm{a}\mathrm{t}\mathrm{i}\mathrm{c}\:\mathrm{i}\mathrm{n}\mathrm{d}\mathrm{e}\mathrm{x}\:\left(\mathrm{I}\mathrm{S}\mathrm{I},\:\mathrm{\%}\right)=\frac{\mathrm{I}\mathrm{W},\:\mathrm{g}}{{\mathrm{W}}_{60\:\mathrm{d}\mathrm{a}\mathrm{y}},\:\mathrm{g}}\times\:100$$

### Sampling

Prior to sampling, the fish were withheld from feed for 24 h to ensure their digestive tracts were cleared. Fifteen fish from each tank were chosen (total *n* = 45 per treatment across three replicate tanks), anesthetized using 100 mg/L MS-222 (Sigma-Aldrich, USA), and then subjected to blood collection. Blood was extracted from the caudal vasculature using non-heparinized syringes. After clotting for approximately 30 min, the samples were centrifuged at 3,500 g for 5 min. at 4 °C. The obtained serum was immediately frozen in liquid nitrogen and stored at − 80 °C for future biochemical and enzyme assessments. Serum biochemical indicators such as glucose (GL 13 20), total protein (TP 20 20), albumin (AB 10 10), calculated globulin, cholesterol (TC 20 10), triglycerides (TG 20 11), alanine aminotransferase (ALT, AT 10 34), aspartate aminotransferase (AST, AT 10 45), urea (UR 21 10), and creatinine (CR 12 50), were quantified using Bio-Diagnostic kits (Egypt) according to the producer’s procedure. Cortisol levels were assessed using an enzyme-linked immunosorbent assay (ELISA) kit (CalBiotech, catalog no. CO368S, USA).

The digestive tracts were carefully excised (*n* = 9 fish/tank; total *n* = 27 per treatment), washed with PBS (pH 7.5, 1 g/10 mL), and homogenized at 4 °C. The samples were then centrifuged at 5,000 g for 5 min. The collected supernatant was stored at 4 °C for the evaluation of digestive enzyme levels. Protease activity was assessed using a Sigma assay kit, employing casein as the substrate^21^. Levels of lipase (LPS, Cat. No. A054-1-1) and amylase (AMS, Cat. No. C016-1-1) were quantified colorimetrically, with measurements recorded at 420 nm and 660 nm, respectively.

Liver and intestinal tissues (*n* = 9 fish/tank; total *n* = 27 per treatment) were collected. For antioxidant enzyme and lipid peroxidation analyses, a portion of the liver tissue was immediately frozen in liquid nitrogen and stored at − 80 °C until biochemical assessment. Another portion of the liver and intestinal tissues was promptly fixed in 10% neutral-buffered formalin for histopathological examination. Following fixation, the samples were sequentially dehydrated using graded ethanol solutions, cleared with xylene, and fixed in paraffin wax. Tissue sections of 5 μm were prepared using a rotary microtome (RM2035; Leica Microsystems, Wetzlar, Germany). These sections were stained with hematoxylin and eosin (H&E), visualized and imaged using a Leica DM500 microscope equipped with an EC3 camera (Leica, Germany). For intestinal morphometric assessment, digital images were analyzed using ImageJ software (Bethesda, MD, USA) following Schneider et al.^23^. Villus height was measured from the villus tip to the villus–crypt junction, villus width at the mid-villus region, and crypt depth from the base of the villus to the muscularis mucosa. Ten well-oriented villi per fish were measured to obtain mean values for each replicate. Hepatic tissue alterations were examined qualitatively to assess structural integrity, melanomacrophage centers, and immune cell infiltration, as described by Gibson-Corley et al.^24^. Five high-power fields (40×) per slide were evaluated blindly by a pathologist.

### Innate immunity

Lysozyme ability was assessed using a microplate assay based on the method of Lygren et al.^25^. In this procedure, 10 µL of serum was added to each well of a 96-well microplate, followed by 190 µL of substrate solution (0.2 mg/mL *Micrococcus lysodeikticus* lyophilized cells, Sigma-Aldrich, USA) prepared in PBS (pH 7.4). The mixture was maintained at room heat with gentle shaking. Detection was measured at 450 nm using an ImmunoMini NJ-2300 microplate reader (System Instruments, Tokyo, Japan) at 1-minute and 5-minute intervals. The relative lysozyme activity (1 Unit) was stated as a 0.001 drop in absorbance per minute. Bactericidal activity (BA) was determined by evaluating the inhibitory effect of serum samples on *Streptococcus agalactiae* growth after a 24-hour incubation in tryptic soy broth (TSB) at 37 °C, with optical density (OD) measured at 570 nm^26^. The BA percentage was calculated using the formula:

$$\:\mathrm{B}\mathrm{A}\:\left(\mathrm{\%}\right)=\frac{\mathrm{O}\mathrm{D}\:\mathrm{c}\mathrm{o}\mathrm{n}\mathrm{t}\mathrm{r}\mathrm{o}\mathrm{l}\:-\:\mathrm{O}\mathrm{D}\:\mathrm{s}\mathrm{a}\mathrm{m}\mathrm{p}\mathrm{l}\mathrm{e}}{\mathrm{O}\mathrm{D}\:\mathrm{c}\mathrm{o}\mathrm{n}\mathrm{t}\mathrm{r}\mathrm{o}\mathrm{l}}\times\:100$$

Neutrophil oxidative radical was valued using the nitroblue tetrazolium (NBT) reduction assay^27^. Briefly, 50 µL of serum samples from each group (*n* = 9) were incubated with 50 µL of NBT solution (0.2% in PBS, pH 7.4) at 25 °C for 30 min. Following incubation, the reaction was terminated by adding 100 µL of acetic acid (30%), and the absorbance (OD) of the resulting blue formazan was determined at 540 nm using an ImmunoMini NJ-2300 microplate reader (System Instruments, Tokyo, Japan).

### Antioxidant assessment

Hepatic antioxidant levels were determined using Nanjing Jiancheng diagnostic kits (Nanjing Jiancheng Bioengineering Institute, Nanjing, China), following the manufacturer’s directions. The specific kits and their protocols were as follows: superoxide dismutase (SOD, A001-3-2) was assessed using WST-1 reagent with absorbance recorded at 450 nm, catalase (CAT, A007-1-1) was evaluated using ammonium molybdate with readings at 405 nm, glutathione peroxidase (GPx, A005-1-2) was determined at 412 nm, and malondialdehyde (MDA, A003-1-1) was quantified using the thiobarbituric acid process with measurements at 532 nm.

### Gene expression evaluation

Liver samples maintained in RNAlater (Sigma-Aldrich, USA) at 4 °C for 24 h were then kept at -80 °C until RNA extraction. Total RNA was isolated using the ABT Purification set (Applied Biotechnology, Cairo, Egypt), and the concentration and purity were judged using a NanoDrop spectrophotometer (BioDrop, Cambridge, UK), with all samples adjusted to a final level of 50 ng/µL. Gene-specific primers (Table 2) were used and RT-qPCR was conducted using SYBR Green. The optimized reaction procedure included reverse transcription at 50 °C for 30 min., primary denaturation at 95 °C for 10 min, followed by 45 cycles of 95 °C for 5 s. and 60 °C for 30 s. *β-actin* was employed as the reference gene, and the specificity of the amplification was verified by performing melt curve analysis. Gene expression levels were determined using the 2^−ΔΔCt^ method.

**Table 2:** Quantitative real-time PCR (RT-qPCR) primer sequences.

Gene	Sequences (5’-3’)	Amplicon size (bp)	Annealing (°C)	Efficiency%	Accession no.
*β-actin*	F: AGCAAGCAGGAGTACGATGAG	135	59	98	XM_003443127.5
	R: TGTGTGGTGTGTGGTTGTTTTG				
*IGF*-1	F: GTGGACGAGTGCTGCTTC	139	58	98	XM_019346352.2
	R: TGCTACTAACCTTGGGTGC				
*SOD*	F: GACGTGACAACACAGGTTGC	198	60	99	XM_003449940.5
	R: TACAGCCACCGTAACAGCAG				
*CAT*	F: TCCTGAATGAGGAGGAGCGA	189	60	96	JF801726
	R: AAACGTGCAAAGTGGCATCC				
*GSH-Px*	F: CCAAGAGAACTGCAAGAACGA	237	58	97	NM_001279711.1
	R: CAGGACACGTCATTCCTACAC				
*IL-1β*	F: TGCTGAGCACAGAATTCCAG	172	60	99	XM_019365841.2
	R: GCTGTGGAGAAGAACCAAGC				
*TNF-1α*	F: CCAGAAGCACTAAAGGCGAAGA	82	60	97	AY428948.1
	R: CCTTGGCTTTGCTGCTGATC				

### Data analysis

Data were evaluated for normality using the Shapiro-Wilk test and for homogeneity of variances using Levene’s test. Once these assumptions were confirmed, a one-way ANOVA was conducted using SPSS software version 20.0 (IBM Corporation, Armonk, NY, USA). Differences among treatment groups were further analyzed using Duncan’s multiple range test at a significance level of *P* < 0.05. The results are expressed as mean ± standard error (*n* = 3 tanks), with each tank mean derived from multiple fish samples (9–15 fish per tank depending on the parameter measured).

## Results

### Phytochemical profile of *Avicennia marina*

**Fig. 1:**
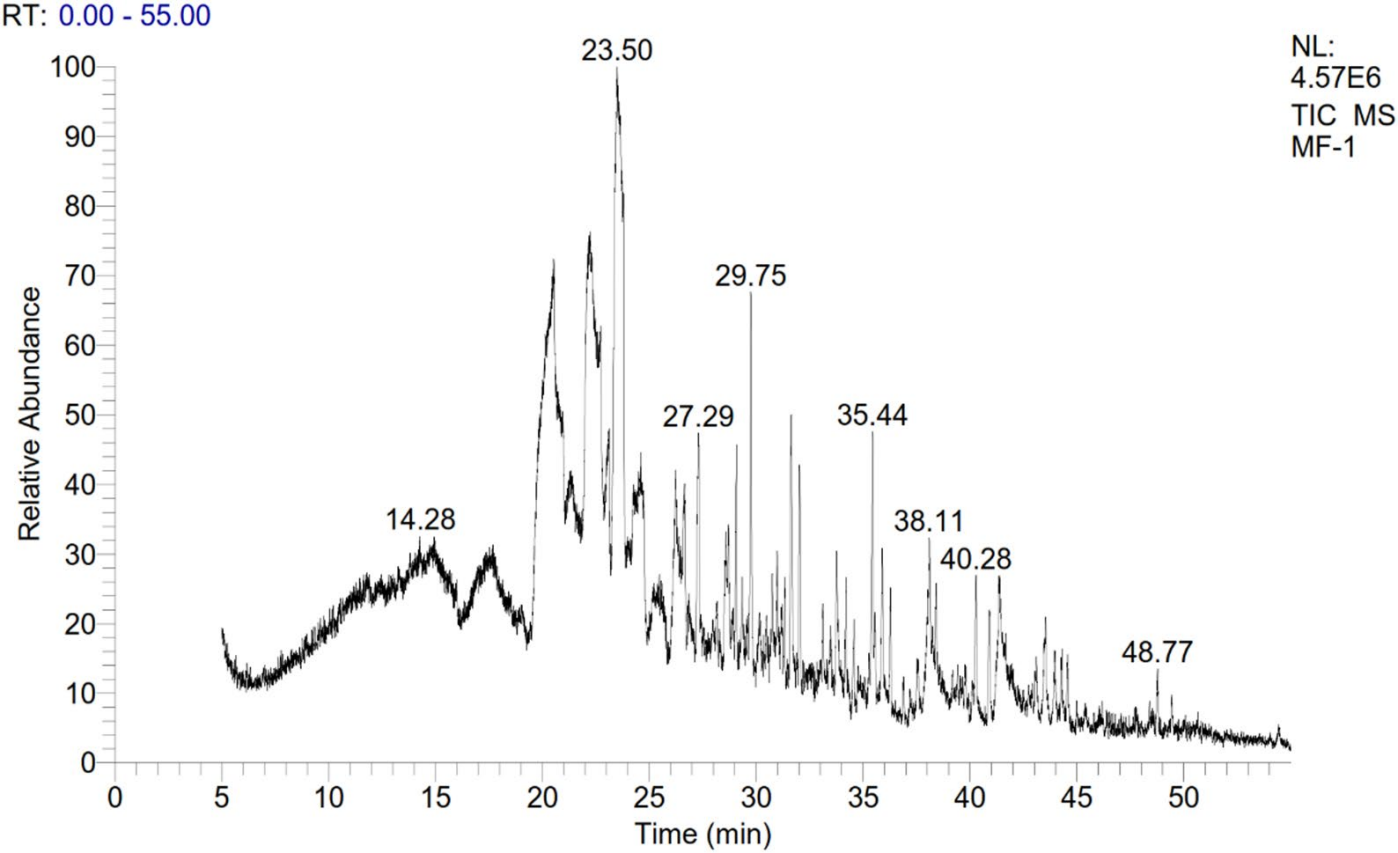
GC-MS Chromatogram of *Avicennia marina* aqueous leaf extract.

The GC-MS evaluation of the aqueous leaf extract of *Avicennia marina* (Table S1 and Fig. 1) revealed a chemically diverse composition, comprising 25 distinct compounds. The major constituent identified was Bicyclo[2.2.2]octan-1-amine (CAS), contributing the highest relative abundance (15.68%). Several saturated and unsaturated long-chain fatty acids and their derivatives were also prominent, including Nonanoic acid (2.65%), Tetradecanal (4.12%), and various octadecenoic acid isomers such as Oleic acid (Z-), cis-13-Octadecenoic acid, and trans-13-Octadecenoic acid (each at 0.87%). Additionally, the profile included notable alkanes such as Pentacosane, Heneicosane, and 17-Pentatriacontene, which are typical components of plant cuticular waxes. The presence of oxygenated compounds like 1-Heptatriacotanol and 2-Heptadecanone, along with heterocyclics such as 1 H-Imidazole-4-menthoal and 6-n-propyl-2,3,4,5-tetrahydropyridine, reflects a complex mixture of potentially bioactive metabolites.

### Performance and biometric indices

**Fig. 2:**
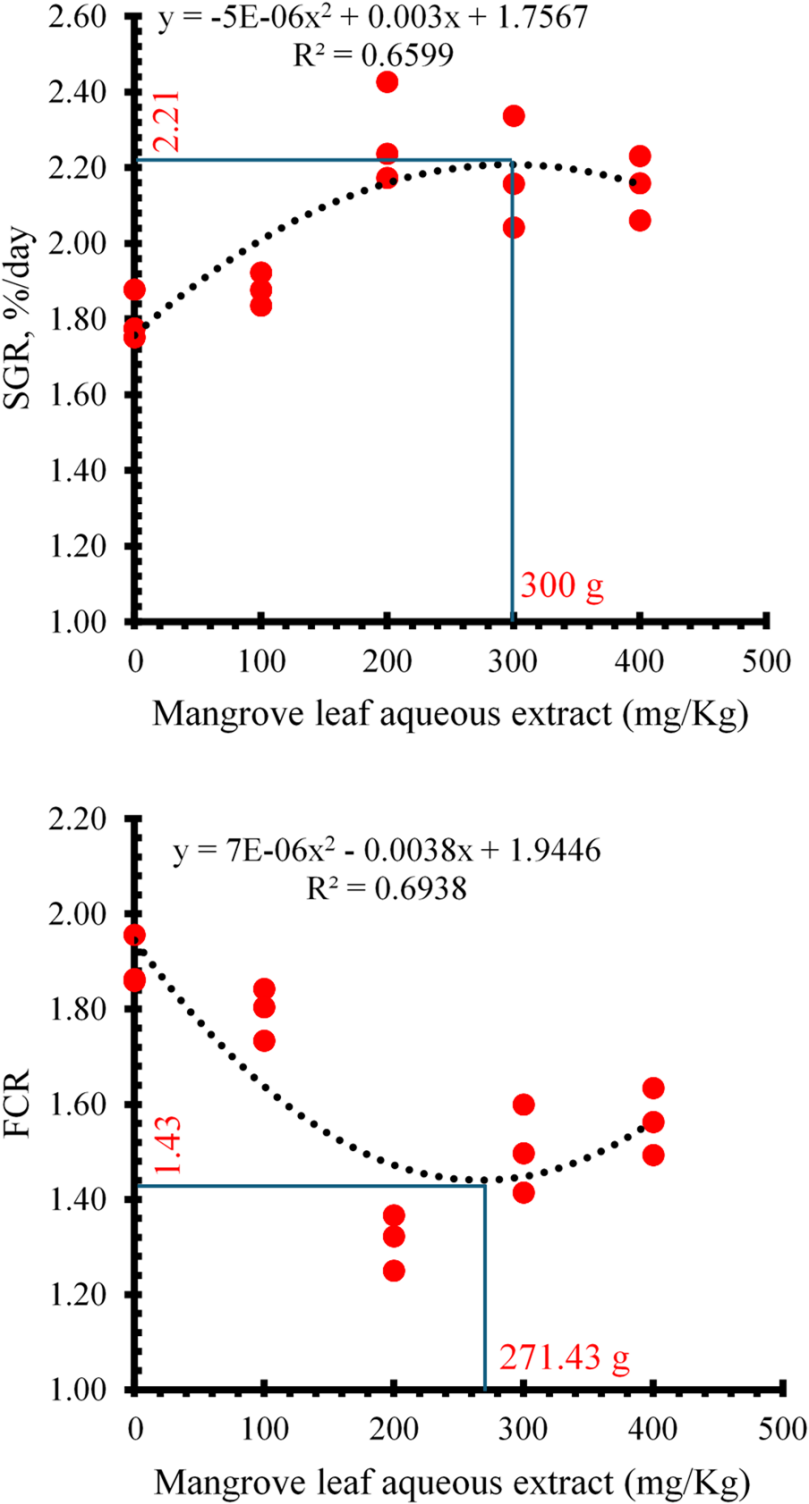
Polynomial regression analysis of the relationship between dietary *Avicennia marina* aqueous leaf extract supplementation and the specific growth rate (SGR) and feed conversion ratio (FCR) of Nile tilapia over a 60-day feeding period.

The dietary inclusion of mangrove leaf aqueous extract significantly influenced growth performance and biometric indices in Nile tilapia (Table 3). Fish fed diets augmented with 200, 300, and 400 mg/kg of the extract exhibited higher BW_60day_ (*P* < 0.05) compared to the control and 100 mg/kg groups, with the 200 mg/kg treatment achieving the highest weight gain (20.01 ± 0.31 g) and weight gain % (293.38 ± 18.28%). This enhancement was mirrored in the specific growth rate (SGR), where fish in the 200 mg/kg group attained the highest SGR (2.28 ± 0.08%/day), significantly surpassing the control and 100 mg/kg treatments. Conversely, the FCR was markedly improved in fish receiving 200 mg/kg (1.31 ± 0.03) followed by the 300 and 400 mg/kg groups, while the control and 100 mg/kg groups exhibited the least favorable FCR values. Survival rates remained consistently high with no differences observed across all treatments (*P* > 0.05). Regarding biometric indices, HSI, ISI, VSI, and K displayed non-significant fluctuations (*P* > 0.05), although values remained within a healthy physiological range. Polynomial regression assessment of SGR and FCR displayed an optimal dietary inclusion amount of *Avicennia marina* aqueous extract between 271.43 and 300 mg/kg to maximize growth and feed utilization in Nile tilapia (Fig. 2).

**Table 3:** Evaluation of growth and biometric parameters in Nile tilapia after a 60-day feeding trial.

Parameters	Mangrove leaf aqueous extract (mg/Kg)				
	0	100	200	300	400
Initial body weight, g	5.2 ± 0.06	5.2 ± 0.06	5.1 ± 0.15	5.13 ± 0.22	5.1 ± 0.15
Final nody weight, g	15.33 ± 0.25 ^c^	16.05 ± 0.21 ^c^	20.01 ± 0.31 ^a^	18.95 ± 0.29 ^b^	18.52 ± 0.24 ^b^
Weight gain %	194.99 ± 6.92 ^b^	208.71 ± 4.71 ^b^	293.38 ± 18.28 ^a^	270.69 ± 19.28 ^a^	263.66 ± 10.74 ^a^
Specific growth rate (SGR, %/day)	1.80 ± 0.04 ^b^	1.88 ± 0.02 ^b^	2.28 ± 0.08 ^a^	2.18 ± 0.09 ^a^	2.15 ± 0.05 ^a^
Feed conversion ratio (FCR)	1.89 ± 0.03 ^a^	1.79 ± 0.03 ^a^	1.31 ± 0.03 ^c^	1.5 ± 0.05 ^b^	1.56 ± 0.04 ^b^
Survival rate (SR, %)	97.78 ± 1.28	98.52 ± 1.48	98.52 ± 0.74	99.26 ± 0.74	98.52 ± 0.74
Hepatosomatic index (HSI, %)	3.53 ± 0.47	4.21 ± 0.46	4.7 ± 0.42	4.94 ± 0.13	4.98 ± 1.38
Intestino-somatic index (ISI, %)	11.89 ± 0.86	12.69 ± 1.07	10.9 ± 0.62	11.58 ± 1.27	11.84 ± 0.65
Visceral somatic index (VSI, %)	18.94 ± 1	22.86 ± 1.97	18.95 ± 0.33	18.95 ± 0.53	21.9 ± 1.33
Fulton’s condition factor (K factor)	2.59 ± 0.13	2.13 ± 0.05	2.19 ± 0.03	2.42 ± 0.21	2.24 ± 0.23

Values in the same row are presented as mean ± S.E. Superscripts ^a, b,^ and ^c^ indicate significant differences at *P* < 0.05.

### Digestive system efficiency and health

Dietary supplementation with mangrove leaf aqueous extract significantly influenced digestive enzyme activities and gut morphometry in Nile tilapia (Table 4). Amylase activity was enhanced in the 200 and 300 mg/kg groups (16.13 ± 0.97 and 16.08 ± 1.08 U/mg, respectively), in comparison to the control (13.28 ± 0.33 U/mg). Lipase and protease levels were upregulated in all enriched groups relative to the control (*P* < 0.05), with lipase activity reaching a peak at 200 mg/kg (22.24 ± 0.83 U/mg) and protease at 300 mg/kg (19.52 ± 0.99 U/mg). In terms of intestinal histomorphology, villus height was markedly increased in fish receiving 200 mg/kg (430.8 ± 4.55 μm), followed by those fed 300 and 400 mg/kg diets, all higher than the control (*P* < 0.05). Similarly, villus width exposed a substantial increase in the 200 mg/kg group (119.17 ± 9.43 μm). Crypt depth also showed a progressive increase with extract levels, reaching maximum values in the 300 and 400 mg/kg groups (44.91 ± 6.23 and 48.56 ± 3.99 μm, respectively), significantly exceeding the control (30.95 ± 2.41 μm).

**Table 4:** Digestive enzyme activities and intestine morphometry of Nile tilapia (*Oreochromis niloticus*) after a 60-day feeding trial.

Enzyme activity (U/ mg)	Mangrove leaf aqueous extract (mg/Kg)				
	0	100	200	300	400
Amylase	13.28 ± 0.33 ^b^	14.65 ± 0.41 ^ab^	16.13 ± 0.97 ^a^	16.08 ± 1.08 ^a^	15.86 ± 0.96 ^ab^
Lipase	16.14 ± 0.56 ^b^	21.28 ± 1.06 ^a^	22.24 ± 0.83 ^a^	22.23 ± 1.02 ^a^	22.12 ± 0.93 ^a^
Protease	16.37 ± 0.6 ^b^	19.05 ± 0.62 ^a^	19.48 ± 0.87 ^a^	19.52 ± 0.99 ^a^	19.34 ± 0.5 ^a^
**Intestine morphometry**					
Villus height (µm)	249.69 ± 2.56 ^d^	276.26 ± 3.22 ^cd^	430.8 ± 4.55 ^a^	355.62 ± 18.57 ^b^	303.79 ± 5.35 ^c^
Villus width (µm)	72.31 ± 3.53 ^c^	77.59 ± 2.1 ^bc^	119.17 ± 9.43 ^a^	98.3 ± 9.11 ^ab^	86.96 ± 5.56 ^bc^
Crypt depth (µm)	30.95 ± 2.41 ^b^	36.37 ± 2.31 ^ab^	36.42 ± 4.31 ^ab^	44.91 ± 6.23 ^a^	48.56 ± 3.99 ^a^

Values in the same row are presented as mean ± S.E. Superscripts ^a, b,^ and ^c^ indicate significant differences at *P* < 0.05.

**Fig. 3:**
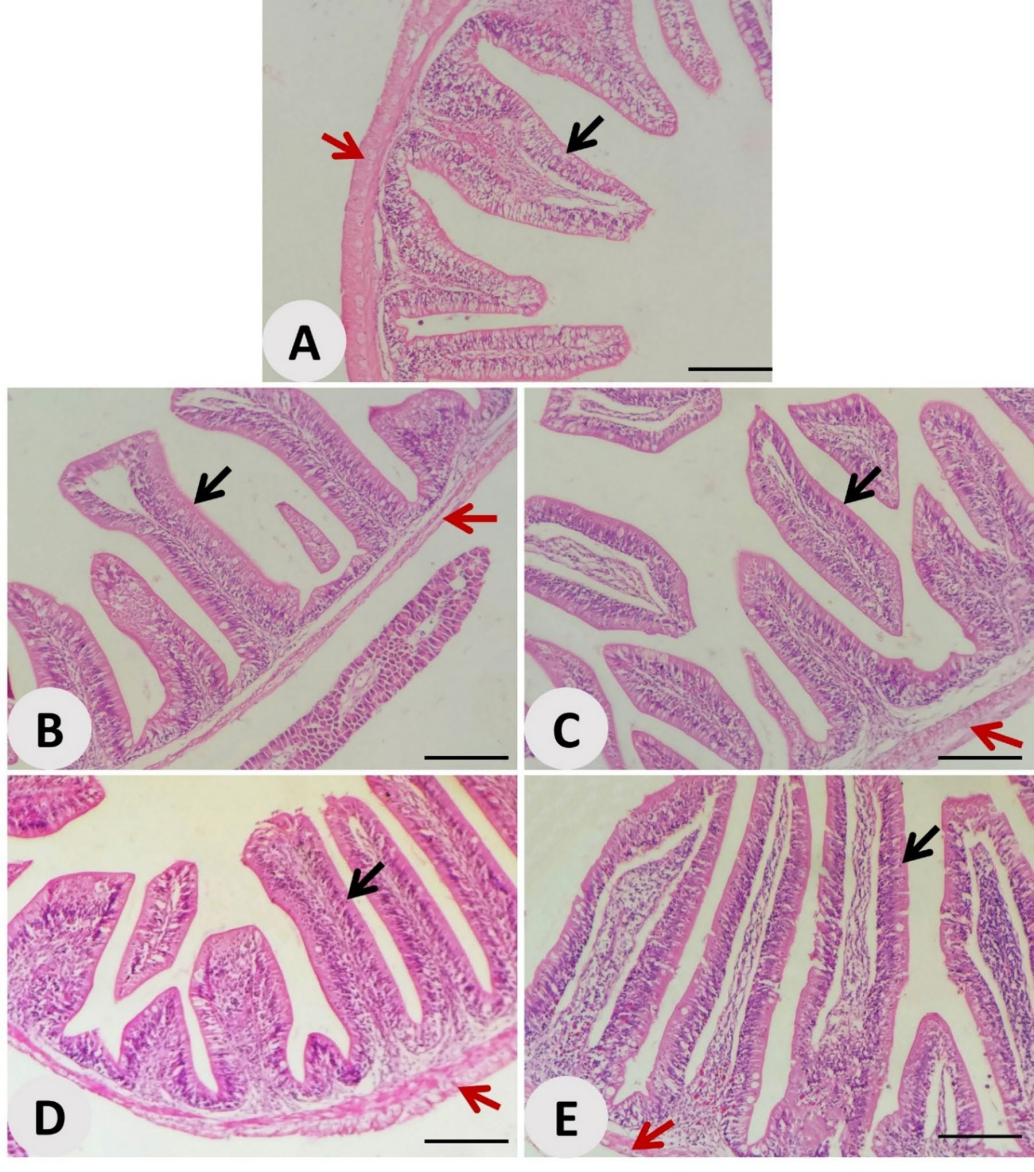
Photomicrographs of Nile tilapia intestine: (**A**) control group, and (**B, C, D, E**) groups supplemented with *Avicennia marina* aqueous leaf extract at 100, 200, 300, and 400 mg/kg of diet, respectively. Black arrowheads denote normal villi, while red arrowheads indicate normal crypt structure and healthy vascularization in the lamina propria. Stain: H&E; Scale bar = 100 μm.

Histological examination of the intestinal tissues in Nile tilapia revealed notable morphological improvements in response to dietary inclusion of *Avicennia marina* aqueous leaf extract (Fig. 3). In the control group (Fig. 3A), intestinal villi appeared relatively short with modest crypt depth and moderate vascularization in the lamina propria. However, progressive enhancements in intestinal architecture were evident in the treatment groups. At 100 mg/kg (Fig. 3B), villi became taller and more organized, with improved crypt integrity and vascularization. These features were further amplified in fish fed 200 mg/kg of the extract (Fig. 3C), where villi appeared elongated and densely packed, suggesting enhanced absorptive surface area and nutrient uptake potential. The 300 mg/kg group (Fig. 3D) exhibited pronounced villus height and width with well-defined crypt structures and intensified vascular profiles, supporting earlier morphometric data indicating structural optimization. Although the 400 mg/kg group (Fig. 3E) maintained villus elongation and vascular health, some crowding and structural compactness were noted.

**Fig. 4:**
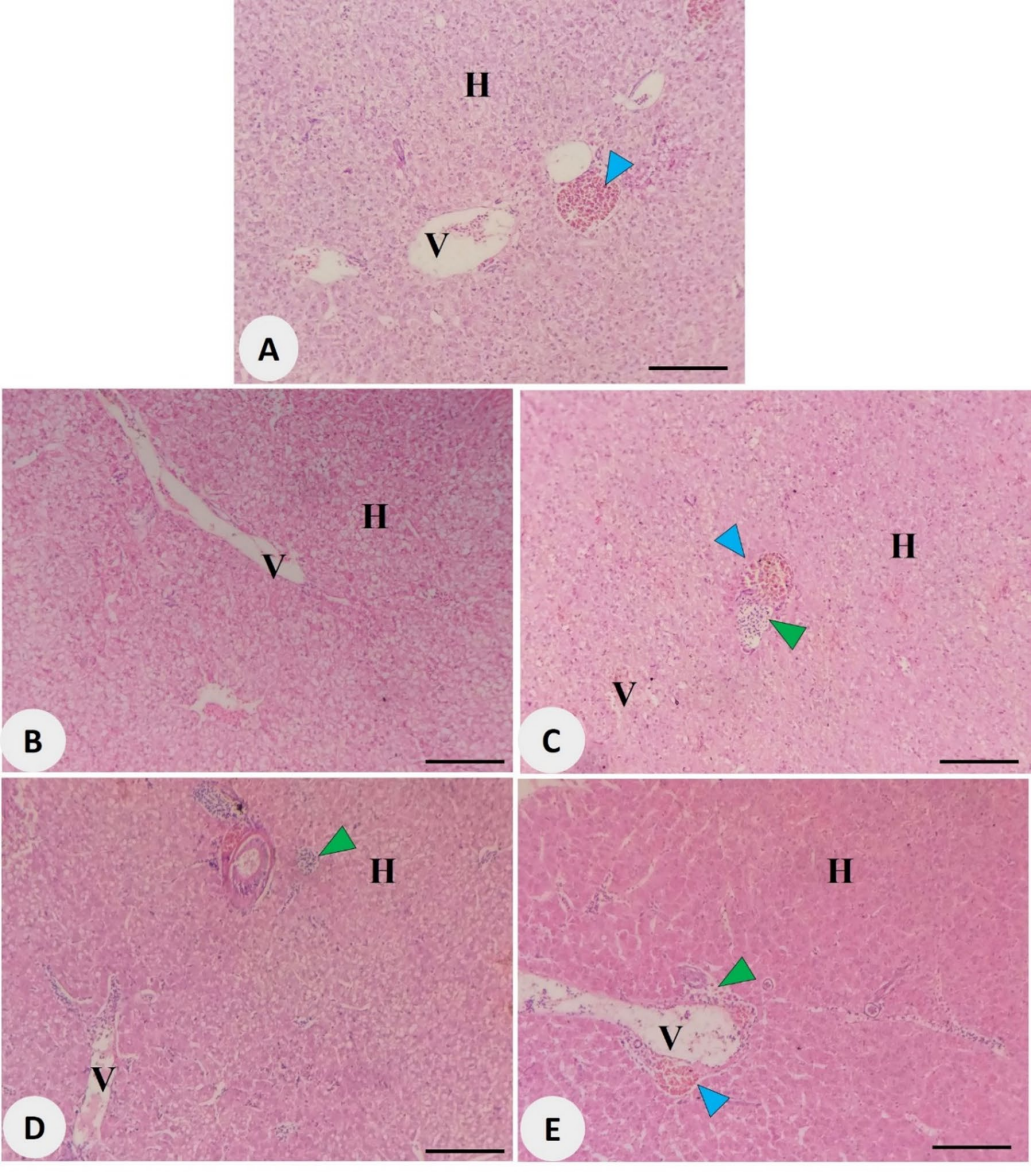
Photomicrographs of Nile tilapia hepatopancreas: (**A**) control group, and (**B, C, D, E**) groups supplemented with *Avicennia marina* aqueous leaf extract at 100, 200, 300, and 400 mg/kg of diet, respectively. Blue arrowheads denote activated melanomacrophages, while green arrowheads indicate regions of immune cell infiltration. H: hepatocyte; V: portal vein; Stain: H&E; Scale bar = 100 μm.

Histopathological analysis of the hepatopancreas in Nile tilapia demonstrated treatment-dependent alterations in tissue architecture and immune activity following dietary supplementation with *Avicennia marina* aqueous leaf extract (Fig. 4). The control group (Fig. 4A) revealed normal hepatic parenchyma with well-organized hepatocytes (H) and intact portal veins (V), accompanied by minimal melanomacrophage centers (blue arrowheads). In contrast, fish fed 100 mg/kg of the extract (Fig. 4B) retained typical hepatic structure with no evident signs of cellular stress or immune activation. However, in the 200 mg/kg group (Fig. 4C), increased melanomacrophage activity and mild immune cell infiltration (green arrowheads) became apparent. These features were slightly more pronounced in fish receiving 300 mg/kg (Fig. 4D), where visible immune cell aggregations were observed around the hepatic parenchyma, yet hepatocyte integrity remained intact. The 400 mg/kg group (Fig. 4E) exhibited both elevated melanomacrophage centers and localized infiltration of immune cells near the portal veins. Despite these changes, no degenerative or necrotic features were detected in any treatment group, suggesting that the extract, even at the highest tested dose, did not induce hepatotoxic effects.

### Blood chemical profile

Serum biochemical analysis of *O. niloticus* after 60 days of dietary supplementation with *Avicennia marina* aqueous leaf extract revealed notable improvements in several health-related parameters (Table 5). Total protein levels were increased (*P* < 0.05) in fish fed 200, 300, and 400 mg/kg diets compared to other groups. Albumin and globulin concentrations also showed significant elevations, especially at the highest inclusion level. In contrast, serum glucose and cortisol levels exhibited a dose-dependent decline, with the minimal value observed in the 400 mg/kg group. Similarly, total cholesterol levels significantly decreased in all treatment groups from 200 mg/kg onwards. However, triglyceride levels remained statistically unchanged across all treatments. Furthermore, liver enzyme activities (ALT and AST) and renal function markers (urea and creatinine) did not show considerable alterations.

**Table 5:** Serum biochemical indices of Nile tilapia (*Oreochromis niloticus*) following 60 days of feeding.

Items	Mangrove leaf aqueous extract (mg/Kg)				
	0	100	200	300	400
Total Protein (g/ dL)	3.23 ± 0.06 ^c^	3.54 ± 0.03 ^b^	4.21 ± 0.15 ^a^	4.07 ± 0.07 ^a^	4.19 ± 0.11 ^a^
Albumin (g/ dL)	1.27 ± 0.03 ^b^	1.32 ± 0.07 ^ab^	1.38 ± 0.01 ^ab^	1.3 ± 0.05 ^ab^	1.43 ± 0.03 ^a^
Globulin (g/ dL)	1.96 ± 0.07 ^b^	2.22 ± 0.05 ^b^	2.83 ± 0.14 ^a^	2.77 ± 0.03 ^a^	2.75 ± 0.14 ^a^
Glucose (mg/ dL)	94.25 ± 1.62 ^a^	84.46 ± 1.22 ^b^	77.86 ± 0.82 ^c^	74.7 ± 1.4 ^cd^	72.3 ± 1.92 ^d^
Cortisol (ng/ml)	37.66 ± 0.48 ^a^	32.61 ± 0.98 ^b^	31.04 ± 0.67 ^bc^	30.6 ± 0.79 ^bc^	30.08 ± 0.38 ^c^
Total cholesterol (mg/ dL)	138.28 ± 4.61 ^a^	125 ± 6.98 ^a^	97.9 ± 5.34 ^b^	89.17 ± 4.32 ^b^	93.54 ± 2.1 ^b^
Triglyceride (mg/ dL)	111.94 ± 3.9	119.59 ± 2.68	119.41 ± 3.22	116.6 ± 2.32	122.52 ± 3.52
ALT (IU/ L)	6.15 ± 0.37	6.24 ± 0.33	6.22 ± 0.55	6.53 ± 0.31	6.17 ± 0.27
AST (IU/ L)	41.67 ± 2.03	42.33 ± 2.73	41.67 ± 1.2	44 ± 1.15	43.33 ± 1.2
Urea (mg/ dL)	5.05 ± 0.37	5.45 ± 0.57	5.59 ± 0.38	5.43 ± 0.18	5.09 ± 0.23
Creatinine (mg/ dL)	0.34 ± 0.03	0.35 ± 0.04	0.37 ± 0.06	0.33 ± 0.03	0.33 ± 0.04

Values in the same row are presented as mean ± S.E. Superscripts ^a, b,^ and ^c^ indicate significant differences at *P* < 0.05.

ALT: Alanine Aminotransferase; AST: Aspartate Aminotransferase.

### Innate immunity

**Fig. 5:**
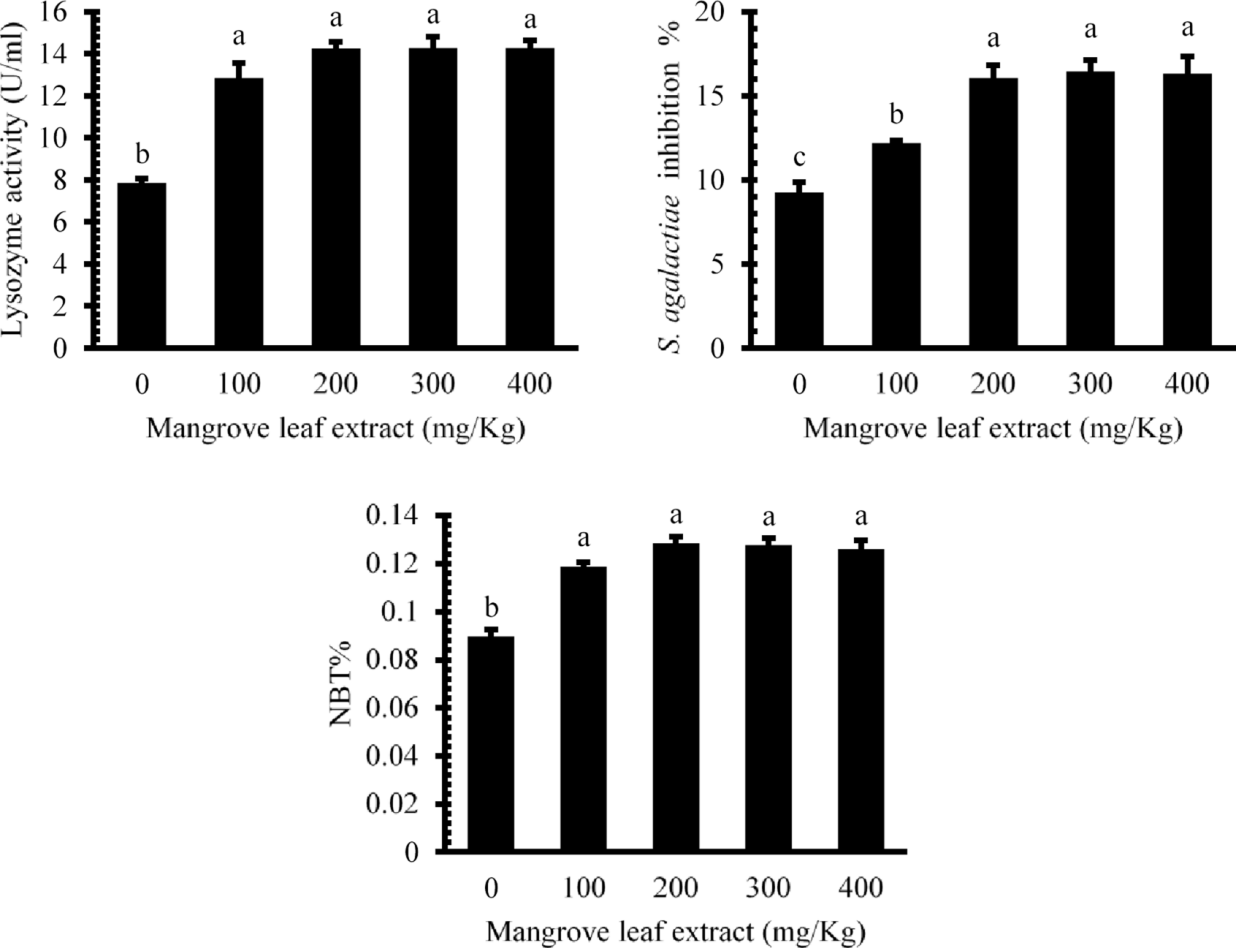
Immune responses in Nile tilapia after 60 days of dietary *Avicennia marina* aqueous leaf extract supplementation.

Dietary supplementation of *Avicennia marina* aqueous leaf extract significantly modulated the innate immune responses in *O. niloticus* (Fig. 5). Lysozyme levels exhibited marked increase in all treatment groups compared to the basal group (*P* < 0.05), with the highest values recorded in fish fed 200–400 mg/kg diets. Similarly, *Streptococcus agalactiae* growth inhibition showed a dose-dependent enhancement, with significant increases observed from the 100 mg/kg group onward, and peak inhibition achieved at 200 mg/kg and higher. Moreover, nitroblue tetrazolium (NBT) reduction was elevated (*P* < 0.05) in all augmented groups compared to the non-supplemented group, with no notable differences among the 100–400 mg/kg treatments.

### Antioxidant enzymes and lipid peroxidation

**Fig. 6:**
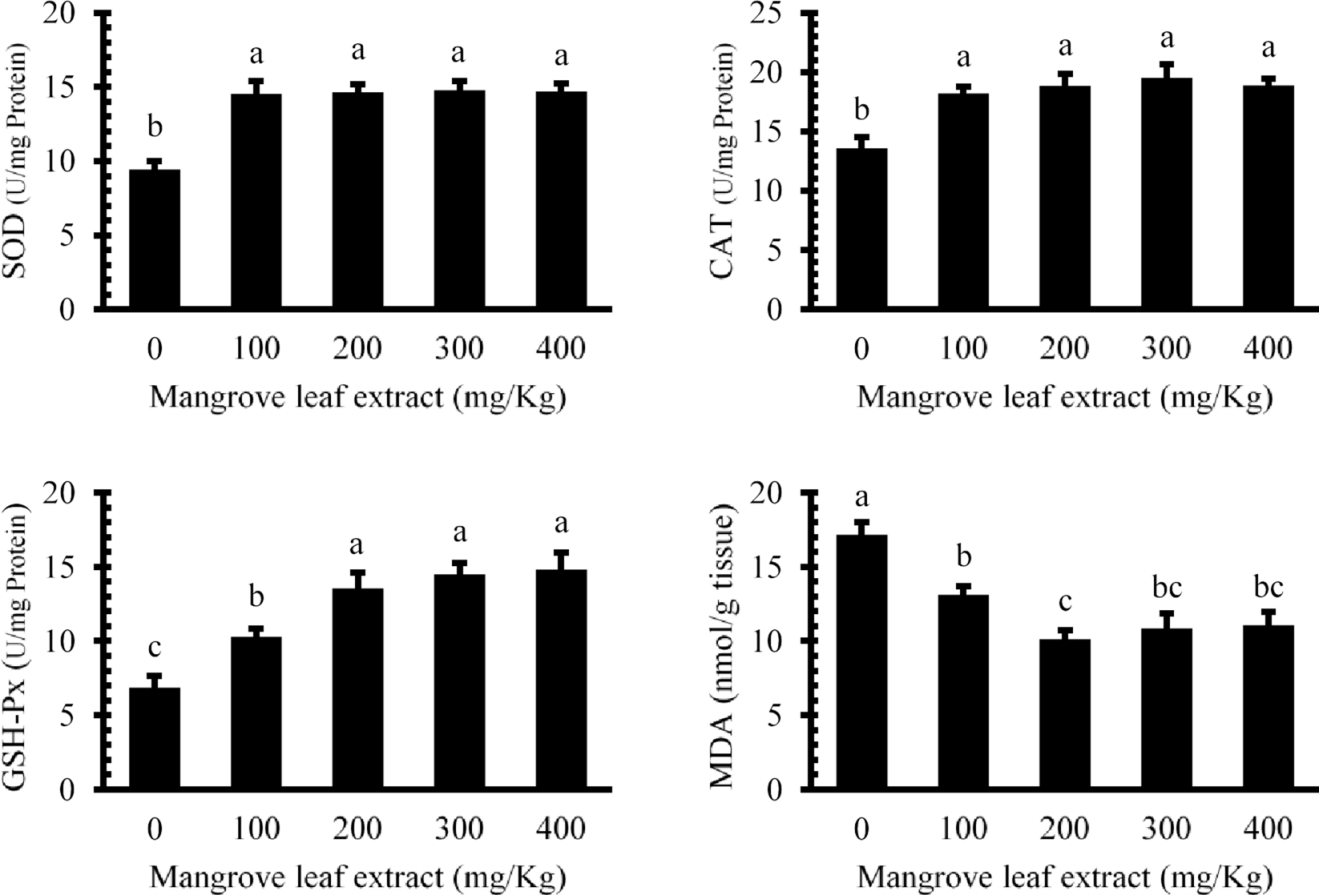
Antioxidant enzymes and lipid peroxidation in liver of Nile tilapia after a 60-day feeding period: SOD (superoxide dismutase), CAT (catalase), GSH-Px (glutathione peroxidase), and MDA (malondialdehyde).

The impact of dietary mangrove leaf aqueous extract on antioxidant enzyme values and lipid peroxidation in the liver of Nile tilapia is shown in Fig. 6. SOD and CAT activities heightened (*P* < 0.05) in all enriched groups compared to the non-treated group. However, no differences were detected among the treated groups. SOD activity increased from 9.41 ± 0.57 U/mg protein in the control group to 14.51–14.75 U/mg protein in the supplemented groups (*P* < 0.05). CAT activity showed a similar pattern, rising from 13.55 ± 0.94 U/mg protein in controls to 18.17–18.87 U/mg protein in treated groups. Glutathione peroxidase (GSH-Px) activity exhibited a significant elevation (*P* < 0.05) in all augmented groups compared to the basal group, with the 200–400 mg/kg groups showing the highest GSH-Px level. GSH-Px activity demonstrated a dose-dependent response, with values of 6.86 ± 0.78, 10.29 ± 0.52, 13.54 ± 1.04, 14.49 ± 0.75, and 14.80 ± 1.14 U/mg protein for 0, 100, 200, 300, and 400 mg/kg diet groups, respectively. Malondialdehyde (MDA) values decreased (*P* < 0.05) in all mangrove groups compared to the reference group. MDA levels decreased from 17.15 ± 0.85 nmol/g tissue in the control to 10.12 ± 0.59 nmol/g tissue in the 200 mg/kg diet group, indicating a 49% reduction in lipid peroxidation, with the lowest MDA value observed at this level. However, no significant differences were found among the 200–400 mg/kg groups.

### Gene expression assessment

**Fig. 7:**
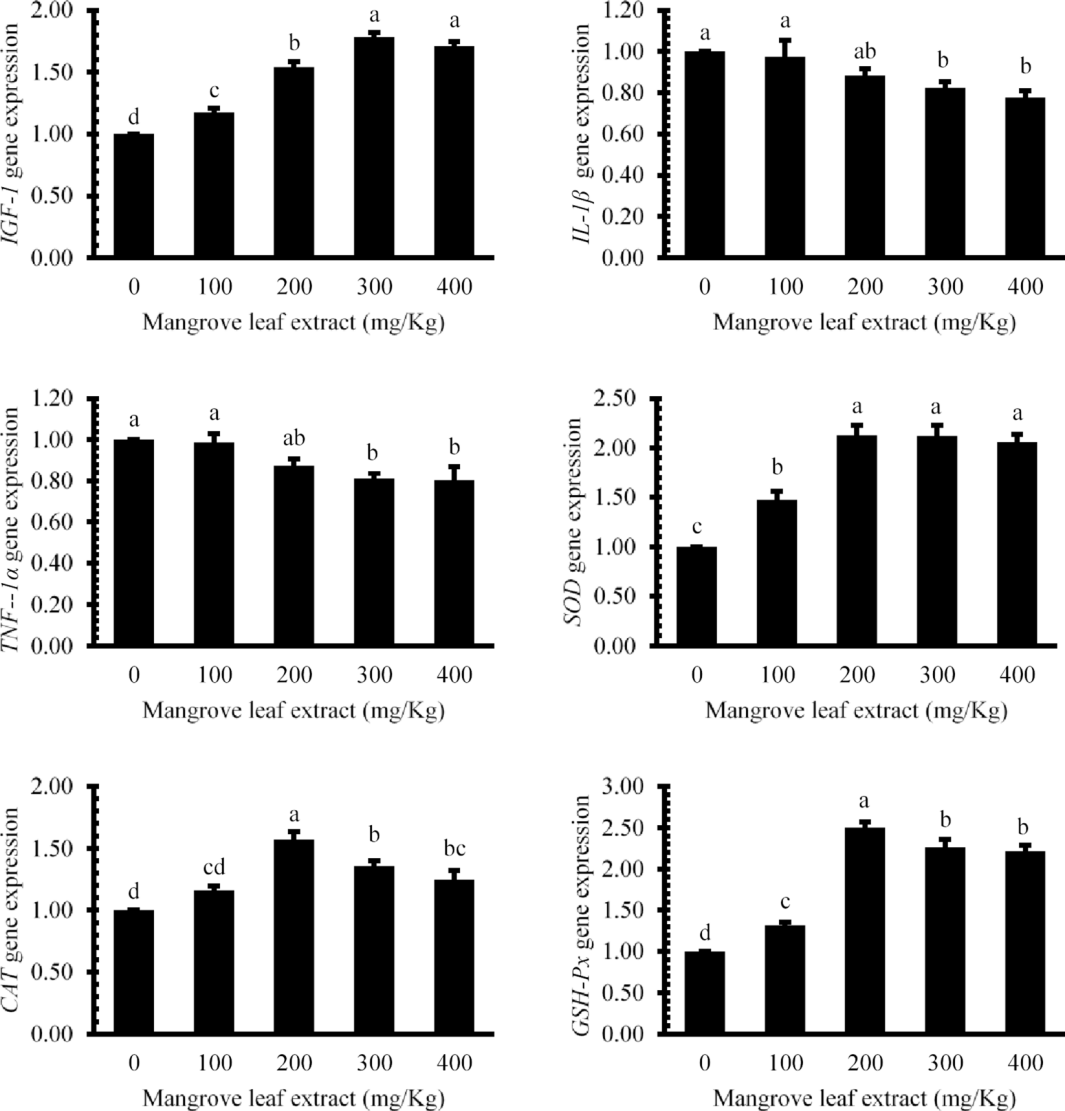
Effects of *Avicennia marina* aqueous leaf extract supplementation on the hepatic expression of growth, immune, and antioxidant-related genes in Nile tilapia (*Oreochromis niloticus*) after a 60-day feeding period. *IGF-1* (I*nsulin-like Growth Factor 1*), *IL-1 β* (*Interleukin-1 Beta*), *TNF-1α* (*Tumor Necrosis Factor Alpha*), *SOD* (superoxide dismutase), *CAT* (catalase), and *GSH-Px* (glutathione peroxidase).

The impacts of dietary mangrove leaf aqueous extract on the hepatic expression of growth, immune, and antioxidant-related genes in Nile tilapia are presented in Fig. 7. The expression of *insulin-like growth factor 1* (*IGF-1*) was elevated (*P* < 0.05) in all treated groups, with the peak expression observed in the 300 and 400 mg/kg groups. *Interleukin-1 beta* (*IL-1β*) and *tumor necrosis factor alpha* (*TNF-1α*) expressions decreased (*P* < 0.05) in the 300–400 mg/kg groups compared to other groups. Regarding antioxidant-related genes, *SOD* expression increased (*P* < 0.05) in all treated groups, with the 200–400 mg/kg groups showing the maximum values. *CAT* and *GSH-Px* expressions were enhanced (*P* < 0.05) in all enriched groups compared to the reference one, with the 200 mg/kg group exhibiting the highest level.

## Discussion

The use of plant-based extracts in aquaculture has attracted growing interest due to their numerous advantages for aquatic organisms, including tilapias. These advantages include boosted immune responses, enhanced antioxidant activity^28,29^, antibacterial effects^30^, as well as improved growth rates and survival^31^. This study emphasizes the broad spectrum of bioactive compounds found in mangrove leaves, 25 distinct compounds, suggests a complex phytochemical composition that could contribute to various physiological benefits in fish. Bicyclo[2.2.2]octan-1-amine as the most abundant (15.68%) known for its amine structure, may exhibit antioxidant and antimicrobial properties^32^. The presence of both saturated and unsaturated fatty acids, including Nonanoic acid, Tetradecanal, and various octadecenoic acid isomers (such as oleic acid), indicates the extract’s capability to provide essential fatty acids. Fatty acids are well-recognized for their role in boosting fish growth performance, improving feed utilization, and modulating immune responses^33^. Specifically, unsaturated fatty acids like oleic acid are recognized for their anti-inflammatory and antioxidative effects, which could directly benefit the antioxidant status observed in fish fed with the extract^34,35^. In addition to fatty acids, the detection of plant wax components such as Pentacosane, Heneicosane, and 17-Pentatriacontene may suggest a protective function, potentially acting as natural stabilizers in the diet matrix^36–38^. Oxygenated compounds like 1-Heptatriacotanol and 2-Heptadecanone may further enhance the extract’s biological activity due to their potential antimicrobial and antioxidant properties^39^. Furthermore, the identification of heterocyclic compounds, including 1 H-Imidazole-4-menthoal and 6-n-propyl-2,3,4,5-tetrahydropyridine, is of interest due to their recognized bioactivity, including antimicrobial, anti-inflammatory, and immune-modulatory effects^40^. These observations are consistent with prior phytochemical analyses of several mangrove species, including *Rhizophora mangle*, *Laguncularia racemosa*^41,42^, *Avicennia officinalis*^43^, *Suaeda maritima*^44^, and others such as *Avicennia marina* and *A. germinans*^32,45–47^. The observed biological activities can be mechanistically linked to the identified compounds. Bicyclo[2.2.2]octan-1-amine (15.68%), the predominant compound, likely contributes to antioxidant defense through free radical scavenging via its amine functional group and may modulate immune cell signaling pathways^32,40^. Unsaturated fatty acids (oleic acid and octadecenoic acid isomers) enhance growth and immunity by serving as eicosanoid precursors for inflammatory regulation, modulating membrane receptor signaling, and activating PPARs that control lipid metabolism and anti-inflammatory gene expression^33,35^. Saturated fatty acids and aldehydes (nonanoic acid, tetradecanal) improve feed palatability and stimulate digestive enzyme secretion through taste receptor activation and cholecystokinin release^48,49^. Heterocyclic compounds (1 H-Imidazole-4-menthoal, tetrahydropyridine derivatives) provide antimicrobial effects and stimulate innate immunity via pattern recognition receptors^40^. Collectively, these compounds likely act synergistically through the Nrf2-ARE pathway (antioxidant defense), NF-κB pathway (immune response), and IGF-1/mTOR axis (growth promotion), consistent with the gene expression patterns observed in this study. Although the present study primarily employed GC–MS profiling to identify volatile and semi-volatile constituents of the *Avicennia marina* aqueous extract, quantitative evaluation of total phenolic and flavonoid contents would provide additional insight into its bioactive potential. Previous phytochemical analyses of *A. marina* have demonstrated substantial concentrations of phenolics, flavonoids, tannins, and saponins, compounds known to contribute markedly to antioxidant, immunomodulatory, and antimicrobial functions in aquatic organisms^9–11,50^. These polyphenolic constituents may act synergistically with the fatty acids, aldehydes, and heterocyclic compounds identified in the current extract to potentiate physiological responses. The lack of quantitative data on phenolic and flavonoid contents therefore represents a limitation that should be addressed in future research to establish clearer structure–function relationships between distinct phytochemical classes and the observed enhancements in growth, immune function, and antioxidant capacity. Nonetheless, the comprehensive GC–MS analysis presented herein provides valuable insight into the extract’s chemical diversity and forms a robust foundation for understanding its multifaceted biological activities in Nile tilapia.

The present work demonstrated that dietary supplementation with *Avicennia marina* leaf aqueous extract heightened the growth status of Nile tilapia, markedly at inclusion levels of 200 and 300 mg/kg. Fish receiving these diets exhibited superior final weight and SGR compared to the non-treated and 100 mg/kg groups. These results align with previous findings indicating that phytogenic compounds derived from mangrove species can positively influence digestion, enhancing metabolism, and stimulating appetite^9,51,52^. The improved feed conversion ratio (FCR) observed at 200 mg/kg further supports the efficacy of *A. marina* in enhancing feed utilization efficiency. The polynomial regression analysis (Fig. 2) revealed optimal inclusion levels of 300 mg/kg for SGR (predicted: 2.21%/day; R² = 0.66) and 271.43 mg/kg for FCR (predicted: 1.43; R² = 0.69), with approximately 66–69% of performance variability explained by extract concentration. The convergence of these optima within 271–300 mg/kg provides a practical recommendation of ~ 285 mg/kg for simultaneously optimizing growth and feed efficiency. The quadratic response reflects classical hormetic effects where moderate doses stimulate beneficial adaptive responses, while excessive levels (> 300 mg/kg) may impose metabolic costs or interfere with nutrient absorption. Similar trends have been reported for other plant-derived additives, where moderate inclusion improved FCR and growth while excessive levels offered no additional benefits or even adverse effects^53,54^. The bioactive compounds identified in *A. marina*, including nonanoic acid, tridecanal, tetradecanal, and bicyclo[2.2.2]octan-1-amine, may contribute to these improvements. Fatty acids and aldehydes are known to enhance energy metabolism and feed palatability, while amine derivatives may stimulate appetite and growth-related hormonal pathways^48,49^.

Despite these performance gains, biometric indices remained unaffected by the treatments. These results suggest that the extract did not induce any pathological alterations or excessive fat deposition, which supports its safety and compatibility as a dietary additive. Similar non-significant changes in biometric parameters were previously reported in tilapia supplemented with other plant-based extracts^55,56^, indicating a physiologically balanced response to functional feed components.

The dietary inclusion of *Avicennia marina* leaf aqueous extract markedly enhanced digestive enzyme activities and intestinal morphometry in *O. niloticus*, supporting its functional role in promoting gastrointestinal health and nutrient utilization. The observed upregulation of amylase, lipase, and protease activities, particularly at 200–300 mg/kg, suggests improved digestive capacity, consistent with prior reports on the stimulatory effects of phytochemicals on enzyme secretion and pancreatic function in fish^57,58^. The peak activities of lipase and protease at 200 and 300 mg/kg, respectively, indicate that specific bioactive compounds in the mangrove extract, such as saturated and unsaturated fatty acids, may directly stimulate digestive enzyme synthesis or improve gut health, thereby optimizing nutrient breakdown and absorption^33^. This enzymatic enhancement is likely a contributing factor to the improved FCR and growth performance discussed earlier.

Corroborating the enzymatic data, significant improvements in intestinal morphology, characterized by enhanced villus height, width, and crypt depth, were observed in the extract-treated groups. These histomorphological enhancements, especially evident at 200–300 mg/kg, suggest improved absorptive surface area and epithelial turnover, which are critical for maximizing nutrient uptake efficiency^59^. The well-defined vascularization and crypt architecture seen in these groups further highlight the trophic effects of the extract on intestinal tissues. These findings are in line with those stated by Yue et al.^60^, who observed similar improvements in gut structure and digestive function following dietary inclusion of botanical extracts rich in flavonoids and fatty acids. The structural compactness noted at 400 mg/kg may indicate a saturation threshold beyond which additional extract does not yield further histological benefits and could potentially disrupt optimal villus spacing. Histopathological analysis of the hepatopancreas revealed treatment-dependent activation of immune components, evident from increased melanomacrophage centers and immune cell infiltration at 200–400 mg/kg, without any signs of tissue degeneration or necrosis. Melanomacrophage centers (MMCs) are known biomarkers of immune surveillance and detoxification in fish, often upregulated in response to immunostimulants or moderate oxidative challenges^61^. Their presence, in conjunction with preserved hepatic architecture, indicates a safe, immunologically active response to the extract rather than a cytotoxic effect.

The current study demonstrated that supplementation with *Avicennia marina* aqueous leaf extract exerted a positive modulatory effect on serum biochemistry, innate immunity, antioxidant defense, and gene expression in tilapia. The elevation of total protein, albumin, and globulin values in the 200–400 mg/kg groups signifies enhanced liver synthetic activity and humoral immune response, aligning with findings by Mansour et al. ^62^, who linked such increases to enhanced health status in fish fed plant-derived immunostimulants. The dose-dependent decline in serum glucose and cortisol, especially pronounced at 400 mg/kg, suggests a reduction in physiological stress, potentially mediated by the antioxidant and adaptogenic properties of the phytochemicals present in the extract^63^. Moreover, the significant decrease in serum cholesterol levels from 200 mg/kg onward may reflect the hypolipidemic potential of bioactive constituents such as phytosterols and polyphenols, which are known to modulate lipid metabolism in fish^64^. Comparable cholesterol-lowering effects have been observed in terrestrial animals supplemented with mangrove, highlighting their potential to enhance lipid metabolism^65,66^. The unchanged triglyceride levels indicate a selective lipid-lowering mechanism, possibly acting more on cholesterol biosynthesis or absorption. The lack of significant changes in ALT, AST, urea, and creatinine values across treatments confirms the hepatic and renal safety of the extract at all inclusion levels, further supported by the histopathological findings. Consistent with our findings, Al-Harthi et al. ^66^ reported no changes in plasma proteins, lipids, or liver enzymes in hens fed mangrove leaves; however, a slight reduction in cholesterol was noted, indicating no harmful effects on metabolic health.

Enhanced lysozyme activity, increased inhibition of *Streptococcus agalactiae*, and elevated NBT reduction across treated groups clearly indicate upregulated non-specific immunity. At the molecular level, dietary inclusion of the extract significantly modulated the expression of key growth and immune-related genes. The increased expression of *IGF-1* in treated fish, especially at 300–400 mg/kg, corroborates the observed growth improvements and suggests enhanced anabolic activity and protein synthesis. Concurrent downregulation of pro-inflammatory cytokines *IL-1β* and *TNF-α* in the higher dose groups points to an anti-inflammatory effect, potentially contributing to immune homeostasis and reduced systemic stress. These immunostimulatory effects are likely attributed to secondary metabolites such as flavonoids, alkaloids, and terpenoids in *A. marina*, which have been previously shown to augment innate defenses in various biological systems^50,52^.

The marked upregulation of hepatic antioxidants (SOD, CAT, and GSH-Px), along with the significant reduction in MDA levels, underscores the extract’s efficacy in mitigating oxidative stress. The upregulation of antioxidant genes *SOD*, *CAT*, and *GSH-Px* further supports the biochemical findings, reflecting transcriptional activation of antioxidant pathways in response to dietary phytochemicals. Elevated antioxidant enzyme activity in all treated groups suggests activation of endogenous defense systems, which can protect cellular components from oxidative damage^50,52^. The lowered MDA values, particularly at 200 mg/kg, reflect reduced lipid peroxidation, a key marker of oxidative injury, affirming the antioxidant potential of *A. marina* constituents.

## Conclusions

The dietary inclusion of *Avicennia marina* leaf aqueous extract at 200–300 mg/kg significantly improved growth performance, feed utilization, digestive enzyme activities, and intestinal morphology in Nile tilapia. Additionally, the extract enhanced antioxidant defense by elevating key enzymes (SOD, CAT, GSH-Px) and reducing lipid peroxidation. Immunomodulatory effects were evident through increased lysozyme activity, bacterial inhibition, and downregulation of pro-inflammatory cytokines (*IL-1β*,* TNF-1α*), alongside upregulation of growth and antioxidant-related genes. These results highlight the potential of mangrove leaf extract as a natural, sustainable feed additive to promote fish health and aquaculture productivity without adverse effects on hepatic or renal function. Further research should incorporate positive controls (e.g., vitamin C or commercial immunostimulants) to benchmark the efficacy of *Avicennia marina* extract against established feed additives and explore its long-term effects on reproductive performance and disease resistance under commercial farming conditions. Investigating the molecular mechanisms underlying immunomodulation and antioxidant pathways will deepen understanding of its bioactive compounds. Additionally, studies assessing synergistic effects with other phytogenic additives and evaluating optimal inclusion rates for different fish species could expand its application in sustainable aquaculture practices.

## Supplementary Information

Below is the link to the electronic supplementary material.

Supplementary Material 1

## Data Availability

The data that support the findings of this study are available from the corresponding author upon reasonable request.
