# Supplementary material for: Immunomodulatory, antioxidant, and growth-promoting properties of Avicennia marina leaf extract on Nile tilapia
Source: Sci Rep. 2025 Dec 17;15:43948. doi: 10.1038/s41598-025-30685-z (PMC12711881; doi:10.1038/s41598-025-30685-z)
Supplement: Supplementary file 1 — Supplementary Material 1 [file 41598_2025_30685_MOESM1_ESM.docx]

**Table S1.** GC-MS Compounds in the aqueous leaf extract of *Avicennia marina*

| **No** | **Compound Name** | **Retention Time**  **(min)** | **Relative Area**  **(%)** | **Molecular Formula** | **Mass**  **(Da)** |
| --- | --- | --- | --- | --- | --- |
| 1 | 5-Hexen-1-ol | 22.44 | 1.13 | C₆H₁₂O | 100 |
| 2 | Nonanoic acid | 21.32 | 2.65 | C₉H₁₈O₂ | 158 |
| 3 | Undecanoic acid | 31.8 | 1.48 | C₁₁H₂₂O₂ | 186 |
| 4 | Tridecanal | 30.22 | 2.09 | C₁₃H₂₆O | 198 |
| 5 | Tetradecanal (CAS) | 27.95 | 4.12 | C₁₄H₂₈O | 212 |
| 6 | trans-2-Dodecen-1-ol | 27.22 | 1.51 | C₁₂H₂₄O | 184 |
| 7 | Hexadecanal (CAS) | 30.22 | 2.09 | C₁₆H₃₂O | 240 |
| 8 | 2-Heptadecanone (CAS) | 39.5 | 1.53 | C₁₇H₃₄O | 254 |
| 9 | 13-Octadecenal, (Z)- | 37.36 | 1.61 | C₁₈H₃₄O | 266 |
| 10 | Oleic Acid (9-Octadecenoic acid, Z-) | 23.93 | 1.06 | C₁₈H₃₄O₂ | 282 |
| 11 | Octadecanedioic acid (CAS) | 41.3 | 0.83 | C₁₈H₃₄O₄ | 314 |
| 12 | Pentacosane (CAS) | 20.97 | 1.93 | C₂₅H₅₂ | 352 |
| 13 | Cyclopentane, (4-octyldodecyl)- (CAS) | 20.97 | 1.93 | C₂₅H₅₀ | 350 |
| 14 | Heneicosane (CAS) | 36.13 | 3.1 | C₂₁H₄₄ | 296 |
| 15 | 1-Heptatriacotanol | 29.33 | 1.06 | C₃₇H₇₆O | 536 |
| 16 | 17-Pentatriacontene (CAS) | 32.1 | 0.65 | C₃₅H₇₀ | 490 |
| 17 | Docosyloxy(tert-butyldiphenyl)silane | 47.87 | 0.66 | C₃₈H₆₄OSi | 564.93 |
| 18 | Bicyclo[2.2.2]octan-1-amine (CAS) | 25.3 | 15.68 | C₈H₁₅N | 125 |
| 19 | 1H-Imidazole-4-menthoal | 22.62 | 2.72 | C₄H₆N₂O | 98 |
| 20 | Sulfurous acid, cyclohexylmethyl heptyl ester | 45.33 | 1.24 | C₁₄H₂₈O₃S | 276 |
| 21 | 6-Octadecenoic acid, (Z)- | 49.36 | 0.87 | C₁₈H₃₄O₂ | 282 |
| 22 | cis-13-Octadecenoic acid | 45.45 | 0.87 | C₁₈H₃₄O₂ | 282 |
| 23 | trans-13-Octadecenoic acid | 49.36 | 0.87 | C₁₈H₃₄O₂ | 282 |
| 24 | 1,2-Butanediol, 1-(2-furyl)- | 22.62 | 2.72 | C₈H₁₂O₃ | 156 |
| 25 | 6-n-propyl-2,3,4,5-tetrahydropyridine | 22.62 | 2.72 | C₈H₁₅N | 125 |
